# Evaluation of the Microcirculation in a Rabbit Hemorrhagic Shock Model Using Laser Doppler Imaging

**DOI:** 10.1371/journal.pone.0116076

**Published:** 2015-02-25

**Authors:** Zhenchun Luo, Pengfei Wang, An Zhang, Guoqing Zuo, Yuanyi Zheng, Yan Huang

**Affiliations:** 1 Department of Critical Care Medicine, The Second Affiliated Hospital of Chongqing Medical University, Chongqing, China; 2 Department of Ultrasound, The Second Affiliated Hospital of Chongqing Medical University, Chongqing, China; 3 Department of Anesthesiology, The Second Affiliated Hospital of Chongqing Medical University, Chongqing, China; 4 Department of Internal Medicine, The traditional Chinese medical hospital of Chongqing, Chongqing, China; National University of Singapore, SINGAPORE

## Abstract

The aim of this study is to evaluate the feasibility of Laser Doppler imaging (LDI) for noninvasive and dynamic assessment of hemorrhagic shock in a rabbit model. A rabbit model of hemorrhagic shock was generated and LDI of the microcirculation in the rabbit ears was performed before and at 0, 30, 60, and 90 min after hemorrhage. The CCD (Charge Coupled Device) image of the ears, the mean arterial pressure (MAP) and the heart rate (HR) were monitored. The mean LDI flux was calculated. The HR of rabbits was significantly (*p* < 0.05) elevated and the MAP was decreased after hemorrhage, compared to the pre-hemorrhage level. Within the initial 30 min after hemorrhage, the perfusion flux lineally dropped down. In contrast, the MAP values did not differ significantly between the time points of 0 and 30 after hemorrhage (*p* > 0.05). Both the flux numbers and the red-to-blue color changes on LDI imaging showed the reduction of the microcirculation. LDI imaging is a noninvasive and non-contact approach to evaluate the microcirculation and may offer benefits in the diagnosis and treatment of hemorrhage shock. Further studies are needed to confirm its effectiveness in clinical practice.

## Introduction

Hemorrhagic shock is a life-threatening condition resulting from decreased blood volume due to blood loss, leading to reduced tissue perfusion, cellular hypoxia and organ damage. Disorders in the microcirculation are linked to the pathophysiology of hemorrhagic shock [1]. In clinical practice, assessment of the status of the microcirculation is of importance in treating patients with hemorrhagic shock. Many factors such as drug delivery and blood volume have been identified to affect the microcirculation [2,3].

There are currently several methods available for assessment of the microcirculation. The microcirculation in the mesentery is commonly evaluated by micro-photography. However, its inherent invasive property hampers its clinical use. In addition, this method does not directly measure the volume of blood flow. Laser Doppler flowmetry (LDF) has been employed to evaluate cutaneous microcirculatory flow, offering continuous perfusion measurement. However, LDF can only examine a small area of perfusion, but fails to give representative data for the surrounding perfusion [4]. Other methods employ some indexes to indirectly reflect the microcirculation, such as heart rate (HR), blood pressure (BP), arterial blood gas, gastric intramucosal pH, and serum lactate [5]. However, these measurements are time-consuming and insensitive.

Recently, Laser Doppler imaging (LDI) has been developed as a new method for assessment of the microcirculation under superficial skin [6]. This method analyzes the Doppler frequency shift of reflected laser beam to detect the blood flow volume [7,8]. LDI has two major advantages over the LDF technique. The first is that blood flow is measured over an area rather than at a single site, obviating the site-to-site variability and improving the reproducibility. Secondly, the laser bean is non-contact, as opposed to the single-probe, which involves direct contact with the skin and may influence blood flow via pressure and movement artifacts [4].

Due to the noninvasive property, good repeatability and relatively large scanning region, LDI has been widely used to investigate the changes of the microcirculation in patients with burns and systemic sclerosis [9]. However, to the best of our knowledge, there are few reports on the application of LDI in assessing the microcirculation associated with shock. A recent study has reported the early evaluation of the microcirculation in hemorrhagic pigs using LDI [10]. Their objective was to determine the ability of LDI for the early detection of hemorrhage and they focused on the “early detection201D and “hemorrhage". While in clinic practice, the “monitoring” of the microcirculation changes in “hemorrhage shock” is more important.

Thus, the aim of this study is to evaluate the feasibility and efficiency of LDI for noninvasive and dynamic assessment of hemorrhagic shock using a rabbit hemorrhagic shock model.

## Materials and Methods

### Rabbit Model of Hemorrhagic Shock

Ten New Zealand albino rabbits, weighing between 2.5 and 3.0 kg, were purchased from the Experimental Animal Center of Chongqing Medical University (Chongqing, China). All animal experiments were conducted under protocols approved by the Institutional Animal Care and Use Committee of Chongqing Medical University.

A rabbit model of hemorrhagic shock was established as described previously [11,12], with minor modifications. Briefly, animals were anesthetized via intramuscular injection of ketamine (21 mg/kg body weight) and xylazine (2.2 mg/kg body weight). Under anaesthesia, each rabbit underwent a cut-down of the femoral arterial line. A 18G fermoral artery cannula was inserted into the femoral artery and connected with a three-way stopper. One way of the stopper was connected with a physiological signal acquisition system (Modal RM6240, Chendu Instrument Company, Chendu, China) for a continuous monitor of heart rate, systolic blood pressure (SBP), diastolic blood pressure, and mean arterial pressure (MAP). The other way was used for the withdrawal of blood from the femoral artery. After the right femoral artery had been cannulated, each rabbit was injected with heparin (3 mg/kg body weight) and allowed to stabilize for 15 min. The rabbits were bled at a rate of 12–18 mL/kg/min for 2–3 min until the blood pressure reached to 45–50 mmHg. The total amount of blood withdrawn was recorded.

### LDI Imaging

**Fig 1:**
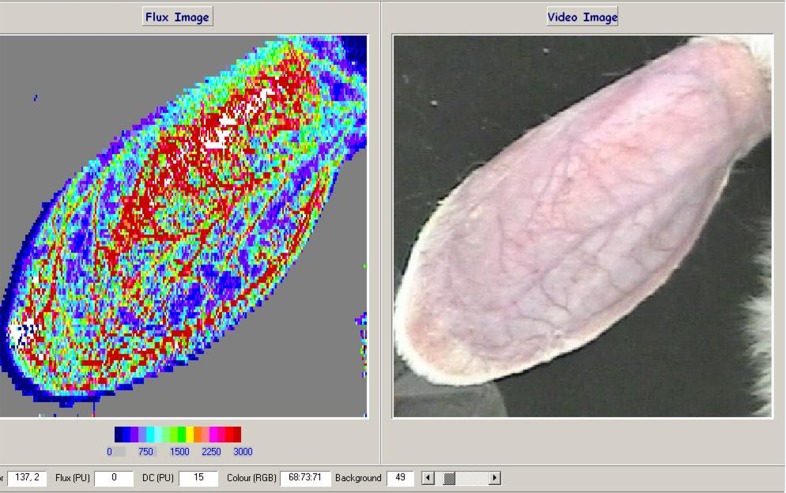
A: LDI image of the rabbit ear (Red, high perfusion; blue, low perfusion). The flux scale is displayed below each image. B: CCD image of the rabbit ear. The two images are shown at the same scale for comparison.

The ear of the rabbits was cleared of the hair. The laser beam, 1 mm in diameter and 785 nm in wavelength and 2.25 mW in output, was scanned across the ear of animals in two dimensions before and at a 30-min interval up to 90 min after withdrawing blood samples. Each scan took about 90 s. The non-contact scanner was positioned 50 cm above the targeted zone, as depicted in Fig. 1. Scans were saved, and mean perfusion in flux units was calculated using the commercial software (moor V5.3, Moor Instruments Ltd., Axminster, UK). The site chosen for the measurement of the microcirculation flux was between the large vessels.

### Statistical Analysis

All data were expressed as mean ± standard deviation. Statistical analysis was performed using one-sample t-test analysis with Microsoft Office Excel 2003 (Microsoft, Redmond, WA, USA). *P*-values < 0.05 were considered significant.

## Results

### Evaluation of the Model of Hemorrhagic Shock

Nine of the 10 animals were continuously bled until shock occurred. The remaining 1 animal showed hypotension with the MAP of 16 mmHg after a loss of 28% of the total blood volume. Hemorrhage was thus discontinued in this animal. The basal HR and MAP level was 148 ± 15.5 bpm and 114.26 ± 9.21 mmHg, respectively. The mean HR at 0, 30, 60, and 90 min after hemorrhage was 192 ± 21.7, 196 ± 25.2, 215 ± 17.2, and 221 ± 20.2 bpm, respectively. The mean MAP at 0, 30, 60, and 90 min after hemorrhage was 42.92 ± 4.63; 40.82 ± 3.35; 29.73 ± 4.12; 24.22 ± 2.23 mmHg, respectively. The mean HR was significantly increased and the mean MAP was decreased compared to basal levels (*p* < 0.05).

### LDI Imaging Findings

**Fig 2:**
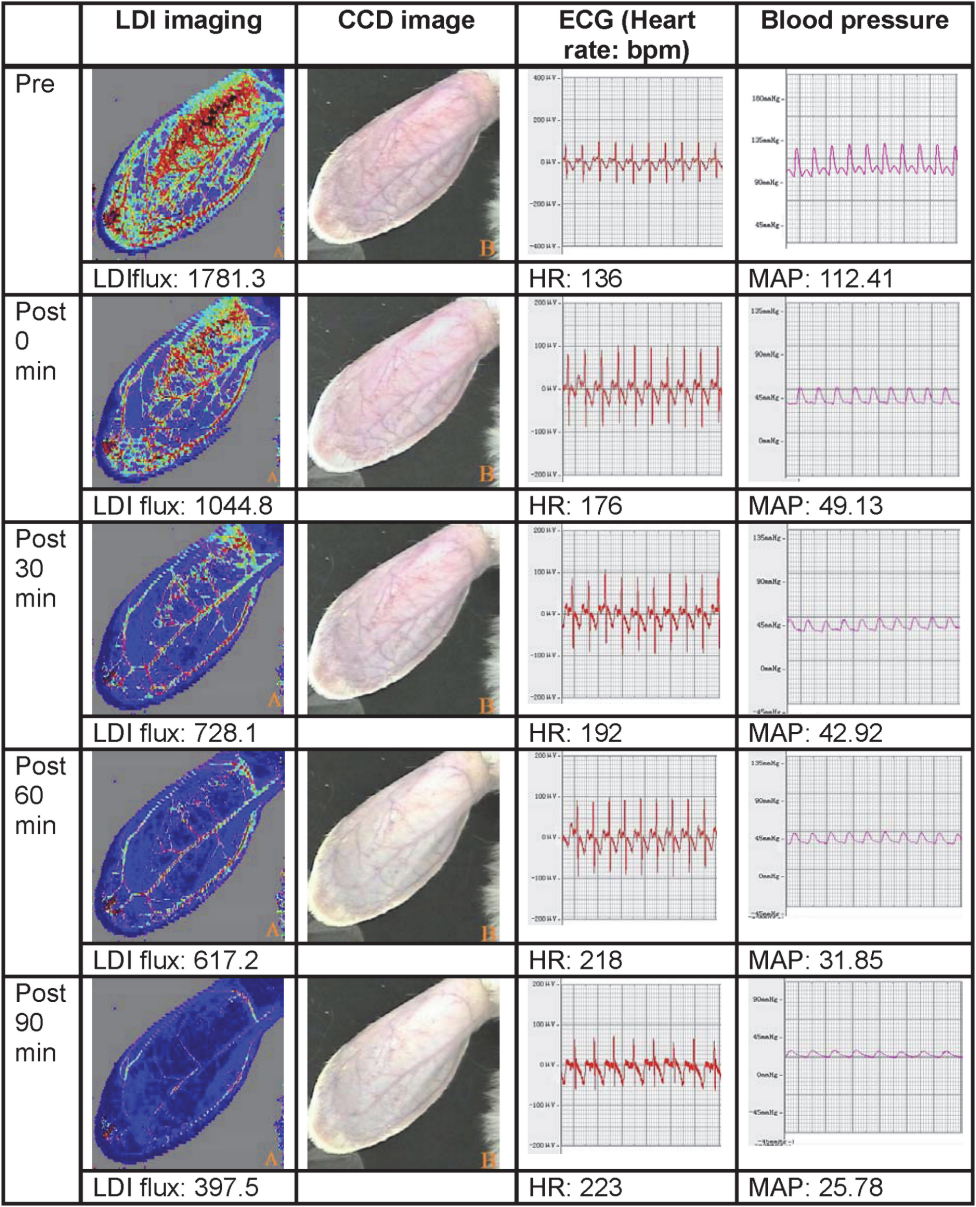
Top-to-bottom LDI scans of a typical rabbit ear at different time points. Lighter shades correspond to higher perfusion in flux units and darker areas correspond to lower perfusion. A mean flux value was obtained for each image.

**Fig 3:**
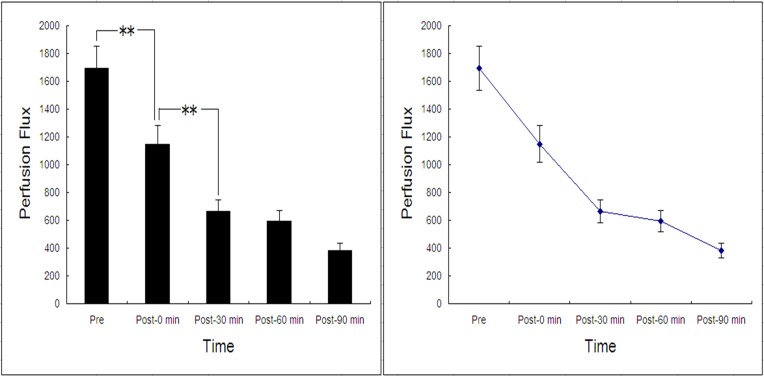
The perfusion flux of rabbit ears at different time points. Boldface indicates statistical significance (*p* ≤ 0.05).

Both the vessels (veins and arteries) and the microcirculation in the ears of the rabbits, which can not been seen by eyes, were readily identified on LDI imaging before hemorrhage (Fig. 1). The perfusion flux was markedly reduced after hemorrhage, decreasing from the basal level of 1691.81 ± 158.22 to 382.1 ± 51 at the time point of 90 min. Both the flux numbers and the red-to-blue color changes in the ears of the rabbits showed clearly the reduction of the microcirculation (Figs. 2 and 3).

**Fig 4:**
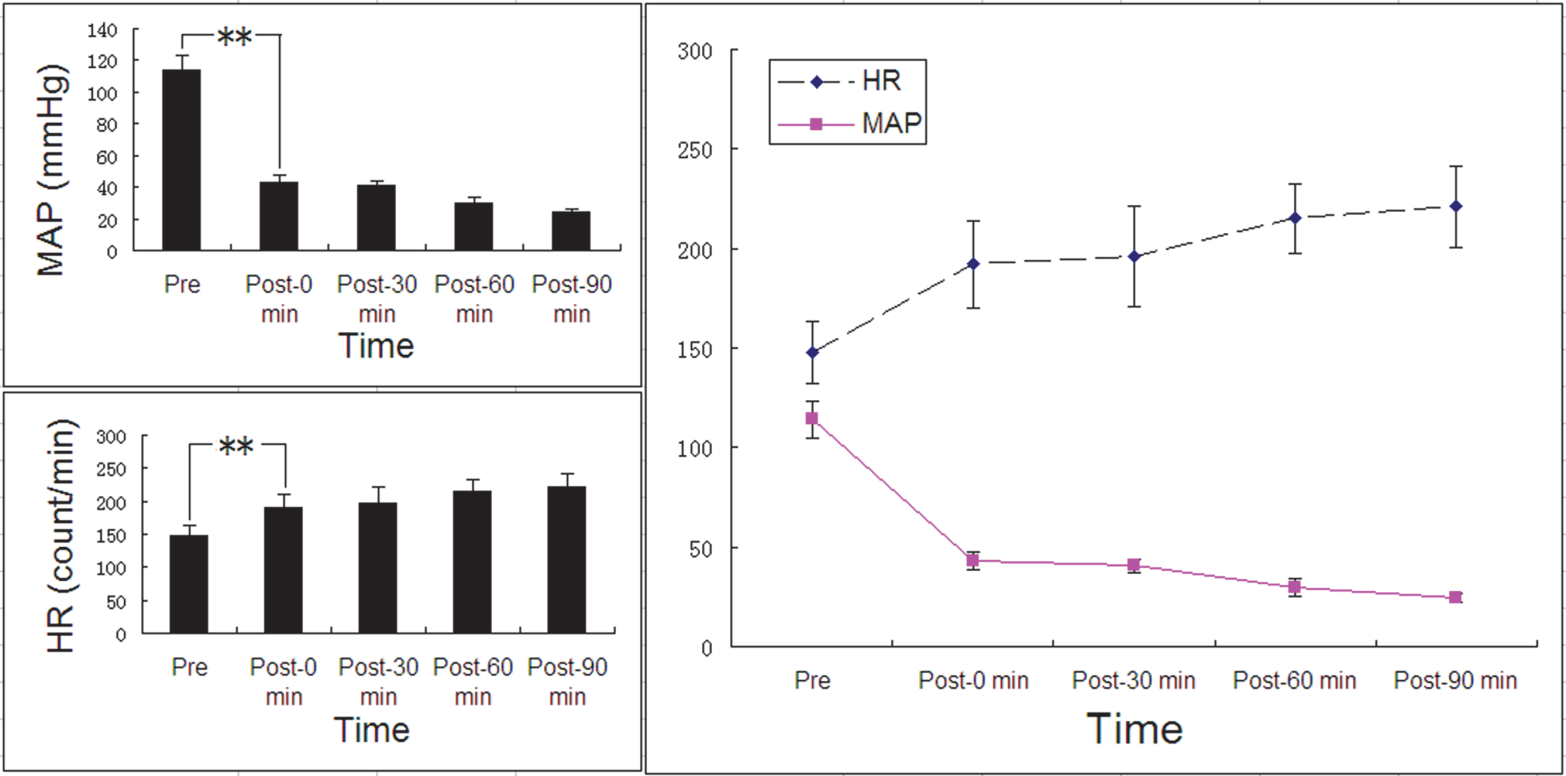
Mean HR and MAP at different time points. Boldface indicates statistical significance (p ≤ 0.05).

Within the initial 30 min after hemorrhage, the perfusion flux lineally dropped down (Fig. 3). In contrast, the MAP had a modest and transient decline immediately after bleeding and the mean values did not differ significantly between the time points of 0 and 30 (*p* > 0.05; Fig. 4).

## Discussion

Our present study showed that the microcirculation in rabbit ears could be well displayed on LDI imaging. During induction of hemorrhagic shock, the color of the rabbit ears changed from red to blue on LDI imaging. Accompanying the color changes, the perfusion flux and blood pressure were decreased and the HR was elevated. Additionally, local microvessel contraction was observed after hemorrhage. These findings suggest a successful establishment of hemorrhagic shock model in rabbits.

LDI imaging data showed that immediately after the withdrawal of blood, there was a sharp decrease in the perfusion flux in the microcirculation of rabbit ears. This finding could be explained by that hemorrhagic shock resulted in a rapid release of catecholamine, which in turn caused a constriction of arterial and venule vessels and reduction of functional capillary density [13].

The decrease of the microcirculation perfusion has been suggested to confer protection to pivotal organs by increasing their blood perfusion [14]. Notably, we found that the perfusion flux decreased linearly during the first 30 min after hemorrhagic shock. In contrast, the MAP and HR level remained relative stable. These observations showed that LDI imaging is a sensitive approach to evaluate the microcirculation in the setting of hemorrhagic shock.

However, some limitations of the LDI imaging method should be noted. First, the maximum imaging depth of this method is less than 1.5 mm, which limits its application to the microcirculation in deep organs or tissues. Second, the use of anesthetics may have impacted basal cutaneous vasomotor reactivity. The influence of drugs used for the treatment of hemorrhagic shock on LDI imaging data is also unclear. Future studies are needed to explore the feasibility of LDI imaging in assessing the microcirculation in hemorrhagic shock patients with pharmacological treatment. Finally, the term commonly used to describe blood flow measured by the laser Doppler technique is ‘flux’: a quantity proportional to the product of the average speed of the blood cells and their number concentration (often referred to as blood volume). This is expressed in arbitrary ‘perfusion units’ and is calculated using the first moment of the power spectral density. So, The flux is a relative unit and there is no absolute normal limit for the flux unit. Therefore, it is of significance to monitor the dynamic changes in flux value.

## Conclusion

The LDI imaging method could be used for noninvasive and non-contact evaluation of the microcirculation in the diagnosis and treatment of hemorrhage shock. It may be useful in sparing patients the morbidity of surgery. Further studies are needed to investigate its feasibility in clinical practice.
